# Effects of a Baby Carrier Intervention on Fathers’ Sensitivity, Involvement, and Hormonal Levels: Follow-Up of a Randomized Controlled Study

**DOI:** 10.1080/15295192.2024.2366763

**Published:** 2024-07-01

**Authors:** Annemieke M. Witte, Marleen H. M. de Moor, Martine W. F. T. Verhees, Anna M. Lotz, Marinus H. van IJzendoorn, Marian J. Bakermans-Kranenburg

## Abstract

***Objective.*** Fathers are of great importance for healthy child development. This randomized controlled study investigated the longer-term effects of an intervention using a soft baby carrier on fathers’ observed sensitive caregiving, involvement, and oxytocin and cortisol levels. ***Design.*** First-time fathers were randomly assigned to use a baby carrier (*n* = 41) or baby seat (*n* = 39) and were asked to use the carrier or seat for at least 6 h per week for 3 weeks. Pretest (*M*_*child age*_ = 2.67 months), posttest (*M*_*child age*_ = 3.99 months), and follow-up (*M*_*child age*_ = 8.25 months) father data were collected. ***Results.*** No intervention effects of baby carrier use on fathers’ sensitivity, involvement, and oxytocin or cortisol levels at follow-up emerged. Unexpectedly, fathers in the baby seat condition reported an increase in the amount of time spent with the infant. Fathers’ sensitivity and oxytocin levels decreased over time, while cortisol levels increased over time, irrespective of condition. ***Conclusions.*** This study showed less optimal hormonal levels in fathers over time, suggesting that support during the first months of fatherhood is needed. Furthermore, use of a baby seat may have contributed to fathers enjoying their time with their infant and consequently their involvement in child caregiving.

## INTRODUCTION

Nowadays, most fathers in Western countries spend more time in child caregiving than in previous decades due to economic and socio-cultural developments (Bakermans-Kranenburg et al., 2019). Father involvement and sensitivity are beneficial for children’s socioemotional and cognitive development (Rodrigues et al., 2021; Sarkadi et al., 2008). Given fathers’ roles in fostering positive child outcomes, supporting fathers in their parenting role from early on is important. Experimental evidence indicates that fathers, like mothers, can benefit from participating in intervention programs (e.g., Buisman et al., 2022). In a pre-registered (https://osf.io/6q3t5/) randomized controlled trial (RCT), however, we found no effects of using a soft baby carrier—a device worn by fathers to carry their infant in an inward-facing position on their torso—a week after completion of the intervention (Verhees et al., 2023). Here we aim to investigate the longer-term effects (5 months after the intervention) of using a baby carrier versus an infant seat on fathers’ sensitive caregiving, involvement, and oxytocin and cortisol levels.

No RCTs have examined baby carrier effects in fathers. One study randomly assigned 49 mothers to either using a baby carrier or using a baby seat (Anisfeld et al., 1990). Mothers in the baby carrier condition were more responsive at 3.5 months postpartum, and more infants in the baby carrying condition were securely attached at 13 months. The baby carrier intervention, however, did not significantly improve mothers’ sensitivity. In another study, 33 adolescent mothers with 2- to 4-week-old infants were randomly assigned to use a baby carrier or read to their infant. At 7 months, infants in the carrier condition were more likely to be securely attached (Williams & Turner, 2020), but observing infant-mother attachment security at 7 months is uncommon and the sample size was small. Importantly, both studies focused on mothers from low socioeconomic backgrounds. RCTs examining the effects of fathers’ using a baby carrier are needed.

Use of a baby carrier may help fathers detect (subtle) infant signals, but fathers may need time to develop sensitive responses. Additionally, fathers may serve as a secure base from which infants explore their environment (Grossmann & Grossmann, 2020), which becomes more important with children’s increased mobility and interest in the environment. Baby carrier effects on paternal outcomes may thus become more apparent after 5 months postpartum. Furthermore, carrying the infant may affect fathers’ oxytocin and lower cortisol levels, mimicking the effects of skin-to-skin contact (Cong et al., 2015; Varela et al., 2018). Even though such effects were not found at posttest in Verhees et al. (2023), “sleeper effects” may still emerge (Goldstein & Bornstein, 2018; Van Aar et al., 2017). Therefore, this study examined the effects of a baby carrier intervention on fathers’ sensitive caregiving, involvement, and oxytocin and cortisol levels 5 months after the intervention, when infants were on average 8 months old.

## METHOD

### Participants

Eighty first-time fathers (*M*_age_ = 33.20, *SD* = 5.36) participated, with a healthy single-born infant (53% boys) around 12 weeks old at pretest. Most fathers (92%) were born in the Netherlands and of Caucasian ethnicity (92%). They worked on average 37 h per week and had 8.28 (*SD* = 1.85) years of education following primary school. Fathers cohabited with the infant and biological mother (see Supplementary Materials and Supplementary Figure 1 for study, sample, and recruitment details).

**Figure 1.**
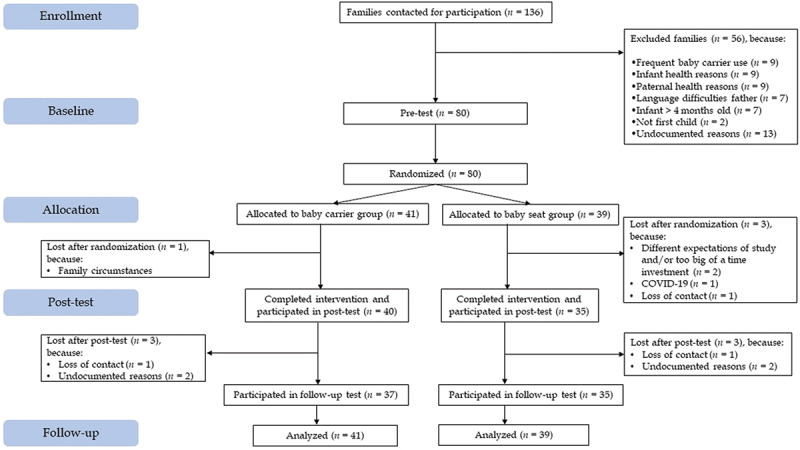
CONSORT flow diagram.

Figure 1 presents a CONSORT flowchart of the study. Randomization to the baby carrier (*n* = 41) or baby seat (*n* = 39) was done after the pretest using a computer-generated randomization sequence. With *N* = 80, power 0.80, alpha .05, and correlations between repeated measures .50, the detectable effect size was Cohen’s *f* = 0.14, corresponding to *d* = 0.28 (G*Power). This effect size is smaller than Anisfeld et al.’s (1990) reported effect sizes, indicating sufficient statistical power.

### Procedures

At pretest, posttest, and follow-up assessments, participants provided saliva samples to measure baseline oxytocin and cortisol levels, then engaged in a 10-min free-play interaction with their infant to measure sensitivity, and provided another saliva sample 10 min after free-play to measure oxytocin and cortisol reactivity. Paternal involvement was measured via a smartphone application and questionnaire, and saliva samples for basal oxytocin and cortisol levels were collected at home on two consecutive days.

### Intervention

Both intervention tools were introduced at home. Fathers in the baby carrier condition chose one of two inward-facing carriers (“Kodaki Flip” and “Ergobaby Adapt.” Fathers were requested to be the sole users and were informed that a temperature logger measured their use of it. Fathers in the seat condition received suggestions on how to use the seat (e.g., during play). Both groups were asked to use the tool for at least 6 h per week, spread over a minimum of 4 days, for 3 weeks. This duration was chosen in close consultation with baby wearing consultants. For more details about the intervention procedures see Verhees et al. (2023).

### Measures

#### Paternal Tool Use

During the 3-week intervention period, fathers reported their daily use of the assigned tool. Average use of the tool during this period was 10.16 h (*SD* = 7.50), which was similar in both groups (*p* = .93, *d* = −0.02). According to the temperature logger (recording the temperature of the baby carrier every 5 min), fathers used the baby carrier on average 11.38 h (*SD* = 8.63) during the intervention period. Logger data was strongly correlated with father-reported baby carrier use, *r*(38) = .87, *p* < .001. Fathers reported to have used the assigned tool on average 3.08 h per week (*SD* = 3.78) between posttest and follow-up, without any group difference (*p* = .37, *d* = 0.22).

#### Paternal Sensitivity

Sensitivity was observed during a free-play interaction (5 min without toys, 5 min with toys) and coded using the Ainsworth et al. (1974) scales for Sensitivity and Cooperation. Five coders rated the observations (*ICC* interrater reliabilities = .70–.73). Sensitivity and cooperation scores were correlated at each assessment (*r* = .63–.68) and were averaged into overall scores for paternal sensitivity per assessment.

#### Paternal Involvement

*Smartphone application*. During 7 consecutive days, fathers received six push notifications with questions about the past 15 min: whether they had spoken about, thought about, or communicated with their infant (*Cognitive/affective involvement*), whether they were near the infant (*Accessibility*), whether the infant was awake, and, if so, whether they had interacted (e.g., changed diaper) or communicated with their infant (*Engagement*). Principal component analyses were conducted, and component scores were used in the analyses. *Time spent with the infant*. Fathers reported how many hours they had spent with their child on each day of the preceding week, counting only the time when both father and child were awake. A mean score was calculated.

#### Paternal Hormonal Levels

See Supplemental Materials for details on hormone sampling and analyses. Salivary samples were analyzed in two batches, for which we controlled in the analyses. *Basal oxytocin and cortisol levels (home samples)*. We calculated the area under the curve (AUC) with respect to ground (Pruessner et al., 2003) based on four samples collected at each assessment, using fathers’ smartphone reports of sampling time in the AUC formula. *Oxytocin and cortisol reactivity (laboratory samples)*. Residualized change scores for oxytocin and cortisol were calculated by regressing post-interaction values on pre-interaction values and saving the standardized residuals, which were used in further analyses.

### Analytic Plan

Outliers (|*z*| >3.29) were winsorized. Data were missing completely at random (Little’s MCAR test, *Χ*^*2*^[753] = 777.71, *p* = .31). The missing data were imputed using multiple imputation for both groups separately, imputing 50 times with 100 iterations using predictive mean matching. The imputed datasets were used in all further analyses. Univariate repeated-measures ANOVAs were carried out using the SPSS Mixed Models procedure with effect coding, including a random intercept of person to account for the repeated measures within persons. This procedure allows pooling of *F*-tests and effect sizes across imputed datasets. In case of significance, post-hoc *t*-tests were conducted. Following CONSORT guidelines for RCTs, we did not test for background differences between the groups. We performed equivalence testing (Lakens et al. 2018) to examine whether a lack of statistical significance was attributable to insufficient statistical power.

## RESULTS

Descriptive statistics are presented in Table 1.

**Table 1.** Descriptive statistics.

	Pretest		Posttest		Follow-up	
Measure	*M* (*SD*)	*N*	*M* (*SD*)	*N*	*M* (*SD*)	*N*
**Baby carrier condition**						
Sensitivity	5.02 (1.48)	41	4.74 (1.38)	37	4.13 (1.58)	38
Basal oxytocin levels	50.07 (10.20)	39	45.37 (7.85)	39	47.22 (8.05)	36
Basal cortisol levels	115.98 (71.64)	40	121.96 (79.12)	39	196.71 (81.11)	37
Oxytocin reactivity levels	−0.01 (0.29)	41	0.06 (0.27)	40	−0.01 (0.15)	39
Cortisol reactivity levels	−0.08 (0.53)	40	−0.02 (0.51)	40	−0.24 (1.02)	39
Involvement (application)	0.13 (0.82)	37	0.11 (1.03)	38	−0.04 (0.78)	29
Involvement (self-reported)	5.99 (2.89)	37	5.33 (1.87)	34	5.78 (1.71)	36
**Baby seat condition**						
Sensitivity	5.18 (1.29)	38	5.14 (1.24)	33	4.63 (1.34)	33
Basal oxytocin levels	50.67 (9.84)	35	47.64 (6.93)	30	46.13 (5.23)	34
Basal cortisol levels	111.92 (76.50)	35	131.94 (88.74)	30	183.78 (85.86)	33
Oxytocin reactivity levels	0.01 (0.36)	38	−0.07 (0.17)	34	0.01 (0.18)	34
Cortisol reactivity levels	0.05 (0.94)	38	−0.05 (0.57)	34	0.22 (1.63)	34
Involvement (application)	−0.11 (1.08)	33	−0.13 (0.96)	33	0.12 (1.01)	26
Involvement (self-reported)	4.65 (1.87)	31	5.27 (2.49)	29	5.71 (1.85)	31

### Paternal Sensitivity

**Figure 2.**
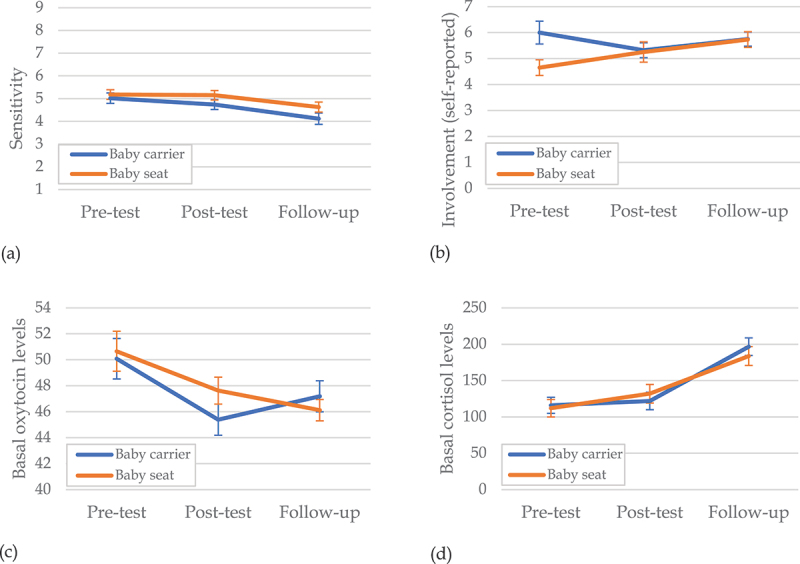
Development of (a) sensitivity, (b) involvement, (c) oxytocin levels, and (d) cortisol levels over time, for the baby carrier and baby seat groups. Error bars display standard errors.

There were no significant effects of condition or condition × time. A statistically significant effect of time emerged, *F*(2,151) = 7.90, *p* < .001, η^2^ = 0.09, with a significant decrease in sensitivity from pretest to follow-up, *t*(73) = −3.74, *p* < .001, *d* = −0.68, but not from pretest to posttest (Figure 2a).

### Paternal Involvement

No significant effects of the condition or time on time spent with the infant emerged. The results indicated a significant condition × time effect, *F*(2,144) = 4.71, *p* = .01, η^2^ = 0.06. Fathers in the seat condition showed a significant increase in self-reported time spent with the infant from pretest to follow-up, *t*(30) = 3.46, *p* < .01, *d* = 1.23, that was not found in the baby carrier condition, *t*(35) = −0.63, *p* = .53, *d* = −0.20 (Figure 2b). For paternal involvement as measured with the application no significant effects were found.

### Paternal Basal Hormonal Levels

For basal oxytocin levels, no significant effects of condition or condition × time were found. A significant effect of time emerged, *F*(2,152) = 7.66, *p* < .001, η^2^ = 0.09, indicating a decrease in basal oxytocin from pretest to follow-up, *t*(75) = −2.90, *p* < .01, *d* = −0.66, as well as from pretest to posttest, *t*(75) = −3.46, *p* < .001, *d* = −0.79 (Figure 2c). Similarly, there were no significant effects of condition or condition × time on basal cortisol levels, but a significant effect of time, *F*(2,152) = 32.31, *p* < .001, η^2^ = 0.30, indicating an increase in basal cortisol from pretest to follow-up, *t*(76) = 7.01, *p* < .001, *d* = 1.59, but not from pretest to posttest (Figure 2d).

### Paternal Hormonal Reactivity

No significant effects of condition, time, or condition × time emerged on oxytocin or cortisol reactivity following free-play.

### Paternal Time Using the Carrier

No significant correlations emerged between time using the baby carrier during the intervention period and follow-up measures (see Supplementary Table 1), although associations with paternal sensitivity, *r*(41) = .22, and time spent with the infant, *r*(41) = .25, were positive and of moderate strength.

### Sensitivity Analyses

Results on effects from posttest to follow-up did not lead to different conclusions.

### Equivalence Testing

The observed effect sizes for differences between the two groups were not different from the lower or upper effect size bound (|*d*| < 0.28), suggesting that for meaningful replicable positive or negative effects, studies with larger sample sizes are needed.

## DISCUSSION

This RCT showed no longer-term effects of using a baby carrier on fathers’ sensitive caregiving, involvement, or hormonal levels. Sensitivity and oxytocin levels decreased while cortisol levels increased over time, irrespective of condition. Fathers in the seat condition reported an increase in the amount of time spent with the infant from pretest to follow-up.

Few studies have examined changes in fathers’ sensitivity over time. One study showed an increase in paternal sensitivity from 12 to 24 months of child age (Hallers-Haalboom et al., 2017), which is later than the present study. Explanations for why fathers decreased in sensitivity remain speculative. In their first year, infants increasingly explore their environment, which may also elicit more controlling and directive fathering behavior. Fathers’ moderate levels of directive behavior combined with positive behaviors, including sensitivity, is referred to as activation parenting (Paquette, 2004). Fathers may need to learn how to sensitively promote infant exploration without becoming directive or controlling.

Fathers’ basal oxytocin levels decreased over time, and their cortisol levels increased. Another study demonstrated that fathers’ oxytocin levels were relatively stable from the prenatal period to 2 months postpartum (Bakermans-Kranenburg et al., 2022). Paternal oxytocin levels may be highest around birth, facilitating father-infant bonding, and thereafter decrease. Increased cortisol levels may be related to balancing work and family life. Fathers need to respond to the demands of caregiving, adjust to changes in their romantic relationship, and accommodate new family routines. At the same time, fathers need to keep up their job performances with restricted nighttime sleep. Our findings point to less optimal hormonal levels in fathers over time, suggesting that support in this stressful time period is needed.

Fathers’ baby carrier use did not lead to enhanced sensitive caregiving. Attachment-based behavioral-focused interventions explicitly promoting sensitive caregiving may be more effective. Promising results from an RCT by Buisman et al. (2022) showed that a video-feedback intervention (VIPP-PRE; De Waal et al., 2022) is effective in enhancing fathers’ sensitive caregiving in the perinatal period. The baby carrier intervention did not affect fathers’ hormone levels either. Of note, oxytocin or cortisol levels were not assessed during or shortly after baby carrying, which may explain differences with reported effects on oxytocin and cortisol levels shortly after skin-to-skin contact (Cong et al., 2015; Varela et al., 2018). Of course, baby carrying differs from skin-to-skin contact, as both the parent and infant wear clothes.

Fathers using the baby seat increased their time spent with their infant. Although a baby seat does not facilitate physical contact, it stimulates face-to-face contact and may contribute to fathers’ enjoying their time with their infant and consequently their involvement in child caregiving. The baby seat may also promote playful interactions, which increase in the course of the first year (Amodia-Bidakowska et al., 2020). If replicated, the results may inspire fathering interventions that stimulate face-to-face contact.

The strengths of the present study include the RCT design with pretest, posttest, and follow-up assessments, the measurement of hormone levels in the home environment (unaffected by travel and stress), and observational measures of paternal sensitivity. However, the study is not without limitations. First, many fathers used the baby carrier less than 6 h per week during the intervention period. Low compliance rates have also been reported in studies promoting skin-to-skin contact (Rheinheimer et al., 2022). Higher compliance rates may result in stronger effects. Second, fathers were from low-risk backgrounds. The effects of using a baby carrier or infant seat may differ in fathers across socioeconomic backgrounds or family constellations.

## IMPLICATIONS FOR THEORY AND PRACTICE

This RCT yielded no effects of baby carrier use on fathers’ sensitive caregiving or involvement at 8 months of the infant’s age nor did use of a baby carrier affect fathers’ basal oxytocin and cortisol levels or hormonal reactivity. Irrespective of condition, sensitivity, and oxytocin levels decreased while cortisol levels increased over time. These findings underscore the need for effective interventions to support fathers in the first year of parenthood.

## Supplementary Material

Supplemental Material

## AFFILIATIONS AND ADDRESSES

Annemieke M. Witte, Vrije Universiteit Amsterdam, Van der Boechorststraat 7, 1081 BT Amsterdam, The Netherlands. Email: http://a.m.witte@vu.nl. Marleen H.M. de Moor is affiliated with Erasmus University Rotterdam, The Netherlands, Martine W.F.T. Verhees with KU Leuven, Belgium, Anna M. Lotz with Leiden University, the Netherlands, Marinus H. van IJzendoorn with University College London, United Kingdom and Monash University, Australia, and Marian J. Bakermans-Kranenburg with University Institute of Psychological, Social and Life Sciences, Portugal, and The New School for Social Research, The United States.

## Data Availability Statement

The data that support the findings of this study are available from the corresponding author, upon reasonable request.
