# Supplementary material for: Effects of a Baby Carrier Intervention on Fathers’ Sensitivity, Involvement, and Hormonal Levels: Follow-Up of a Randomized Controlled Study
Source: Parent Sci Pract. 2024 Jul 1;24(2-3):106–17. doi: 10.1080/15295192.2024.2366763 (PMC11259205; doi:10.1080/15295192.2024.2366763)

**SUPPLEMENTARY MATERIALS**

***Participants***Fathers were recruited via municipal records, infant welfare centers, midwife practices, and social media. One participant was not the biological father of the infant but cohabited with the mother from mid-pregnancy onwards. Exclusion criteria included neurological disorders, endocrine diseases, psychiatric disorders, cardiovascular diseases, use of psychoactive medications, excessive alcohol or drug intake and use of hard drugs within the past three months, MRI contraindications (e.g., metallic foreign objects), using a baby carrier for more than 5 hrs per week at the time of inclusion, or having an injury that could hinder use of a baby carrier. Due to recruitment difficulties, we included 7 participants who met an exclusion criterion (MRI contraindications, *n* = 3; diabetes and use of medication potentially interfering with the endocrine system (metformin), *n* = 1; ADHD, *n* = 1; cardiovascular disease, *n* = 1; infant born after 36 weeks and 6 days of gestation, *n* = 1) (see also Lotz et al., 2021; Lotz et al., 2022; Verhees et al., 2023). Excluding these 7 participants from the analyses did not lead to different conclusions.
 Fathers’ average working hours per week in the three phases of data collection were as follows: pre-test; 37.06 hrs (*SD* = 9.93, range 0–60), post-test; 37.33 hrs (*SD* = 8.98, range 0–60) and follow-up; 37.90 hrs (*SD* = 7.06). Average days of paternal leave were 8.64 days (*SD* = 4.27) in the first two weeks after birth and 6.72 days (*SD* = 5.87) between the second and 10^th^ week after birth.  ***Procedures***Research location was determined by fathers’ preferences and whether they participated in fMRI scanning. Pre- and post-test sessions were conducted either in the lab (82% of pre-test and 76% of post-test sessions) or at home (11% of pre-tests and 15% of post-tests) or partly in the lab and partly at home when the infant was for example sick (6% of pre-tests and 9% of post-tests). All follow-up assessments took place at the fathers’ homes. Fathers received a travel allowance and financial reimbursement for their participation in the study.
 Approximately two weeks after completion of the pre-test (*M* = 13.27 days, *SD* = 4.75), researchers visited the fathers at home to give instructions about the assigned tool (soft baby carrier or baby seat). After this visit, the 3-week intervention period started. The post-test was scheduled, when possible, shortly after the intervention period. Average number of days between the start of the intervention and post-test was 28.04 (*SD* = 12.73, range 20–90 days). For three participants, the post-test could not be scheduled within three weeks after the intervention due to scheduling difficulties (*n* = 2) or measures taken against COVID-19 (*n* = 1). Average number of days between the post-test and follow-up test was 130.04 (*SD* = 25.09, range 42–245). For one participant, follow-up measures were conducted during a later time period due to scheduling difficulties. For one participant, the follow-up test had to be scheduled shortly after the post-test due to the fixed end date of the project. Supplementary Figure 1 shows an overview of the data collected at each timepoint.
 Fathers’ self-reported working hours at pre-test, post-test and follow-up were not correlated with the amount of time fathers used the assigned tool during the three-week intervention period (as measured with the temperature logger and self-reports) or with the average amount of time fathers reported to have used the assigned tool per week in the four months between post-test and follow-up.
 After the intervention period, fathers rated their appreciation of the assigned tool on a scale ranging from 0 (very negative) to 100 (very positive). No difference emerged between appreciation of the baby carrier, *M* = 69.27, *SD* = 31.54, and the baby seat, *M* = 66.03, *SD* = 26.57, *t*(58) = -0.42, *p* = .26, *d* = 0.11.

***Hormone sampling and analyses***For the home samples, fathers were instructed to collect morning saliva samples immediately after awakening, and evening samples right before going to bed, but before they brushed their teeth. They were requested not to eat, drink (except water), chew gum, smoke or physically exercise in the 30 min prior to sampling. Via an application on their smartphones fathers were guided through the saliva collection procedure and they reported on the time of saliva collection and any activities (e.g., eating, drinking) in the 30 min prior to sampling. More than 90% of participants adhered to the home sampling instructions at pre-test, post-test, and follow-up. Participants were instructed to store the samples immediately in their freezer after collection. Samples were picked up by researchers at the participant’s home, transported on ice packs, and stored in -20˚C freezers until analysis. For the lab samples (collected for determination of hormonal reactivity), participants were instructed to refrain from drinking alcohol and excessive physical exercise in the 24 hours before the sessions, from drinking caffeine on the day of the sessions, and from eating, smoking and drinking (except for water) in the 30 min before the sessions.  ***Oxytocin*.** Saliva samples were collected using a cotton swab (Salivettes, Sarstedt). Fathers softly chewed on the swab for 60 sec and moved the swab around in their mouth to stimulate saliva production. Salivary oxytocin samples were analyzed at RIAgnosis (Sinzing, Germany). After centrifugation of the salivettes at 4˚C for 30 min with ca. 5000 *g* centrifugal force, 0.3 ml of saliva was pipetted into a vial. Oxytocin was quantified using radioimmunoassay. The detection limit of oxytocin was 0.1 pg/ml. Inter-assay and intra-assay variability was <10%.  ***Cortisol*.** Cortisol samples were collected by means of passive drooling (SalivaBio, Salimetrics). Participants drooled 1.5 ml saliva into a 2 ml cryogenic vial, either directly or using a straw-like saliva collection aid (SalivaBio, Salimetrics). After collection, samples were immediately stored in a refrigerator at -20˚C until laboratory assessment. Salivary cortisol samples were analyzed at Dresden LabServices GmbH (Germany) using luminescence immunoassay (IBL International GmbH). Twenty µl of saliva was used for the analysis of cortisol. The detection limit for cortisol was 0.012 µg/dl. A random selection of 32% of the pre-, post- and follow-up samples was assayed in duplicate, and the intra-assay coefficient of variation was 6%. Inter-assay variability was computed from controls run at each microtiter plate and amounted to ≤8%. None of the assessments showed significant differences in oxytocin or cortisol levels between fathers who collected saliva samples during weekdays and fathers who collected saliva samples during weekend days.
 In total, 2, 4, and 9 fathers had partly missing basal hormone values at pre-test, post-test, and follow-up, respectively. Incidental missing basal hormone values were imputed using the regression equations predicting evening values from morning values on the same day and vice versa. When both values were missing for one day, we replaced this day’s values with fathers’ values of the other day. ***Imputation***The imputation model included the following variables: fathers’ age at pre-test, fathers’ educational level, infant age at pre-test, infant sex, infant health, pregnancy complications, fathers’ sleep at pre-test, paternal protective parenting at pre-test, time using the assigned tool as measured with the temperature data logger, reported time using the tool during the intervention period, change in paternal depressive symptoms from pre- to follow-up test, reported time using the tool on average per week in the time between post-test and follow-up, and all outcome variables assessed at pre-test, post-test and follow-up. One participant in the baby seat condition withdrew informed consent after participating in the pre-test, and therefore had missing data on all variables except condition allocation. This participant was included in the multiple imputation analyses to adhere to the intention-to-treat principle (Ye et al., 2011).

**Supplementary Table 1.** Pooled correlations between follow-up measures and amount of time using the baby carrier during the intervention period (*n* = 41).

|  | Time using the baby carrier^a^ |
| --- | --- |
| Sensitivity | .22 |
| Basal oxytocin levels | .18 |
| Basal cortisol levels | .18 |
| Oxytocin reactivity levels | .08 |
| Cortisol reactivity levels | .22 |
| Involvement (application) | .16 |
| Involvement (self-reported) | .25 |

*Note.* ^a^Time using the baby carrier according to temperature logger data.

*Supplementary Figure 1.* Overview of data collected at each timepoint. Temperature logger data were collected only for fathers in the baby carrier condition.
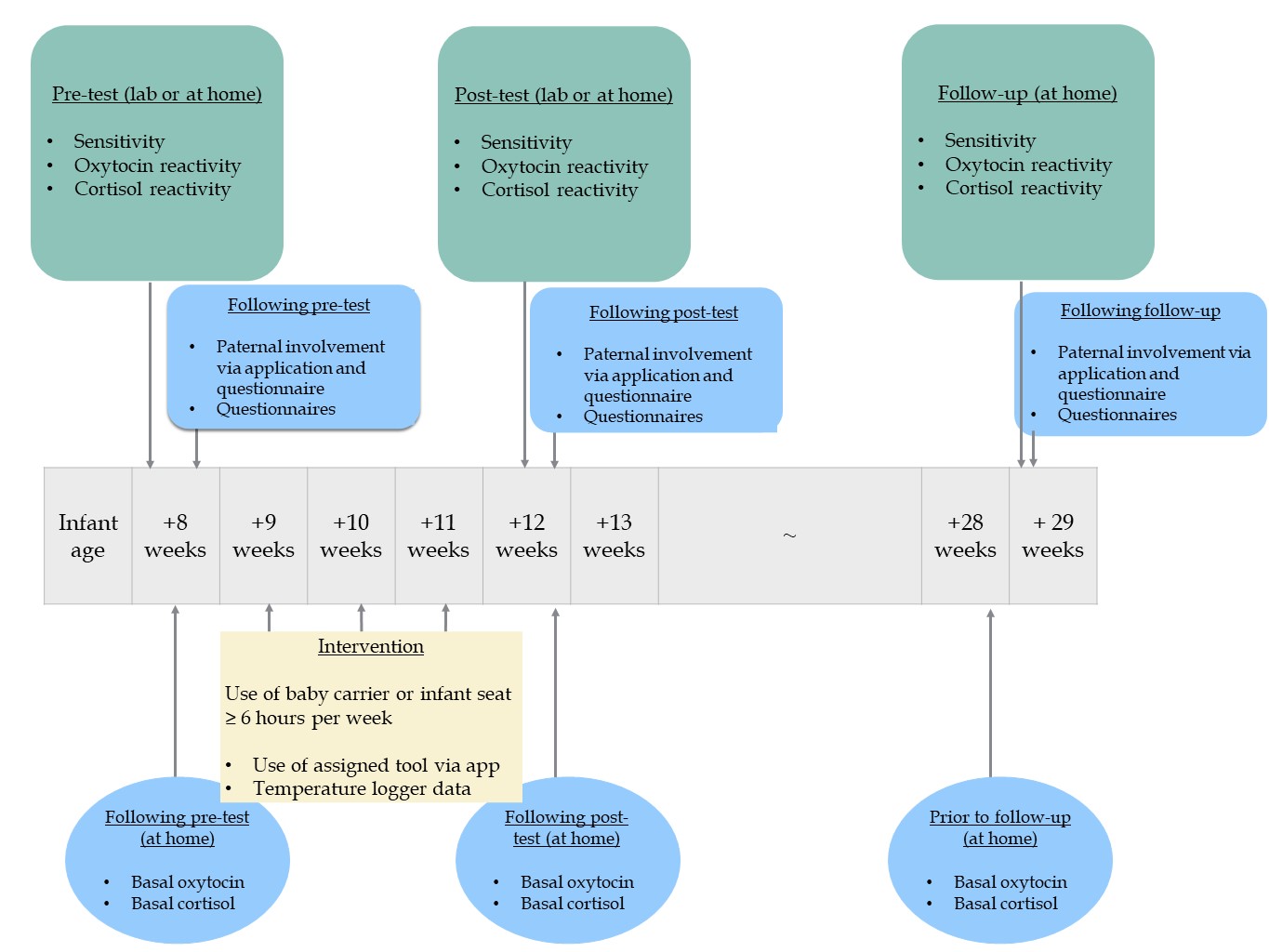

Supplement: Supplemental Material [file HPAR_A_2366763_SM4601.docx]
